# A Case of BRAF-V600E-Positive Pulmonary Pleomorphic Carcinoma Successfully Treated With Dabrafenib and Trametinib Administered via a Percutaneous Endoscopic Gastrojejunostomy Tube for Ileus Caused by Small Intestinal Metastasis

**DOI:** 10.7759/cureus.81586

**Published:** 2025-04-01

**Authors:** Jukiya Higashi, Kotoko Miyoshi, Kazuhiro Hirai, Fumiaki Kanazawa, Hitoshi Nakaji, Aki Miyagaki

**Affiliations:** 1 Department of Respiratory Medicine, Toyooka Public Hospital, Toyooka, JPN; 2 Department of Gastroenterology, Toyooka Public Hospital, Toyooka, JPN

**Keywords:** braf-v600e, dabrafenib, ileus, non-small cell lung cancer, percutaneous endoscopic gastrojejunostomy tube, pulmonary pleomorphic carcinoma, small intestinal metastasis, trametinib

## Abstract

Pulmonary pleomorphic carcinoma (PPC) is a rare subtype of non-small cell lung cancer (NSCLC). It has a rapid progression and poor prognosis and is resistant to conventional chemotherapy.

The efficacy of molecular-targeted drugs for patients with PPC with targetable driver mutations has been reported. However, most molecular-targeted drugs are administered orally, limiting their application in cases where oral administration is difficult.

We report the case of a 78-year-old male patient diagnosed with stage IIA lung cancer. He underwent lobectomy and was pathologically diagnosed with PPC harboring a BRAF-V600E mutation. His lung cancer recurred two months postoperatively with multiple metastases, including those in the small intestine, which caused intussusception and ileus. Because the resected specimen from the small intestinal tumor resembled the histopathological results of the preoperative lung tissue, treatment with dabrafenib and trametinib could be effective.

A percutaneous endoscopic gastrojejunostomy (PEG-J) tube was placed on the anal side of the intussusception site to depressurize intragastric pressure, allowing drug administration while decompressing the stomach. Treatment initiated for a few days improved abdominal symptoms, and computed tomography (CT) revealed tumor shrinkage.

This is the first reported case of a patient with malignant intestinal obstruction successfully treated with targeted therapy drugs administered via a PEG-J tube, which is a viable method for patients with NSCLC with driver mutations who cannot take oral medications or via a nasogastric tube. Furthermore, therapies targeting driver mutations may be effective for patients with PPC.

## Introduction

Pulmonary pleomorphic carcinoma (PPC) is a rare subtype of non-small cell lung cancer (NSCLC), accounting for 0.4-1.6% of malignant lung tumors [1,2]. PPC is traditionally treated with chemotherapy similar to NSCLC, but with a more aggressive clinical course because it is less responsive to cytotoxic agents [3,4]. Reportedly, 8/9 (89%) patients with progressive disease (PD) responded to primary chemotherapy with the combination of carboplatin plus paclitaxel [5]. In recent years, there have also been scattered reports of cases with PPC responding to immune checkpoint inhibitors [6]. However, a universally established standard chemotherapy regimen for this rare histologic entity is unavailable.

Notably, the advent of molecular-targeted therapies has dramatically improved the survival and quality of life of patients with NSCLC with driver mutations [7]. Dabrafenib and trametinib have been shown to work together to inhibit the mitogen-activated protein kinase pathway in BRAF-V600E mutation-positive lung cancer. A phase II study of dabrafenib plus trametinib in 36 untreated patients with stage IV NSCLC and positive BRAF gene V600E mutation reported an overall response rate of 64% and a median progression-free survival of 10.9 months for the primary endpoint [8].

However, studies on the efficacy of molecular-targeted drugs in patients with PPC are limited, and the therapeutic approach is not yet well established [9]. Furthermore, most molecular-targeted drugs are primarily administered orally, so their administration to patients with intestinal dysfunction will be difficult.

We describe the case of a patient with intussusception and ileus caused by small intestinal metastasis of PPC harboring the BRAF-V600E mutation. The patient was successfully treated with dabrafenib and trametinib, administered through a percutaneous endoscopic gastrojejunostomy (PEG-J) tube.

## Case presentation

A 78-year-old male patient with a 25-pack-year smoking history was diagnosed with clinical stage IIA lung cancer in the left upper lobe. Subsequently, he underwent lobectomy combined with chest wall resection. Histopathological examination indicated pleomorphic carcinoma of the lung (pT3N0M0, stage IIB, with chest wall invasion). The tumor was positive for the BRAF-V600E mutation in the AmoyDx pan lung cancer PCR panel, with a 95% tumor proportion score of programmed death ligand 1 (PD-L1) staining.

Two months after the lobectomy, the patient was admitted to the hospital emergently due to abdominal pain and difficulty eating. On admission, appendicitis was suspected initially based on the symptoms, and antibiotic treatment was initiated.

**Figure 1:**
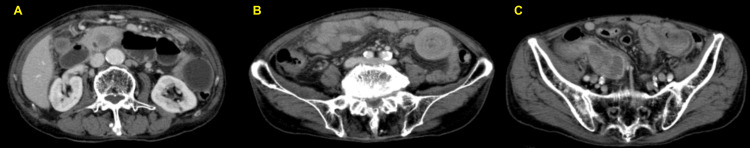
Abdominal CT scan at emergency hospitalization (A) Dilatation of the upper small intestine and mass lesions in the pancreatic head and hepatic flexure, (B) jejunal intussusception, and (C) mass lesions in the appendix. CT: computed tomography

However, follow-up computed tomography (CT) revealed jejunal intussusception; dilatation of the upper small intestine; mass lesions in the pancreatic head, appendix, and hepatic flexure; and multiple enlarged abdominal lymph nodes (Figure 1). The patient had no history of abdominal surgery or use of opioids or other drugs that could cause paralytic ileus, and no electrolyte abnormalities were observed (sodium 140 mEq/L, potassium 3.8 mEq/L, chloride 107 mEq/L, magnesium 2.0 mg/dL, corrected calcium 9.6 mg/dL). Given these findings, the disease course was suspected to be neoplastic.

**Figure 2:**
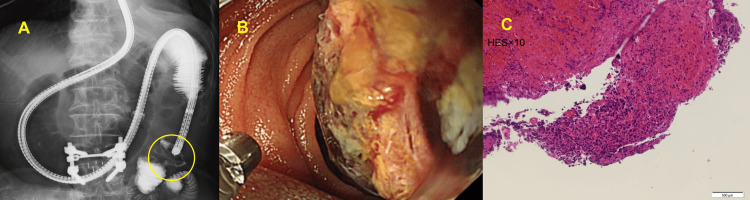
Enteroscopy image showing a mass lesion (A) Intestinal intussusception was located in the jejunum beyond the ligament of Treitz, which was removed by patently advancing the scope, and a mass lesion was observed (yellow circled) in the presenting part. (B) Biopsy was performed at the same site. After biopsy, an ileus tube was placed over the area of intestinal intussusception. (C) Biopsy confirmed metastasis of the pleomorphic carcinoma of the lung.

Enteroscopy was performed to release the intussusception and evaluate the pathology. Endoscopic findings revealed a neoplastic lesion in the upper jejunum with a narrowed lumen (Figure 2). Histology confirmed malignant lesion consistent with metastatic pleomorphic carcinoma of the lung. Following the biopsy, the intussusception was reduced, and an ileus tube was placed. However, the intussusception and ileus repeatedly relapsed and remitted.

Thus, a PEG-J tube was placed on the anorectal side of the intussusception. PEG-J involves inserting and placing a gastrojejunostomy tube into the upper jejunum through the lumen of an existing PEG fistula or PEG catheter. This procedure is performed primarily for nutritional support, medication delivery, and decompression.

**Figure 3:**
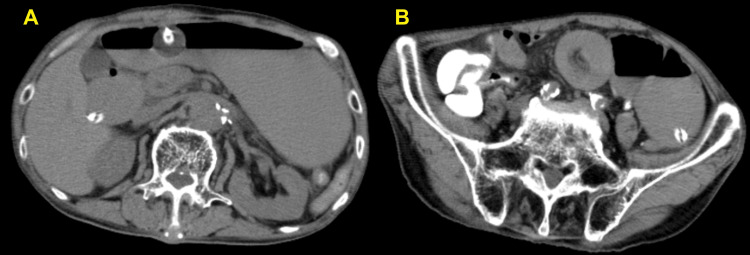
Abdominal CT scan at the day after PEG-J implantation (A) Worsening of ileus and (B) a new jejunal intussusception further anorectally from the catheter tip. CT: computed tomography; PEG-J: percutaneous endoscopic gastrojejunostomy

A day after PEG-J insertion, CT showed a new jejunal intussusception further downstream from the catheter tip. The small intestinal dilatation had worsened, indicating that the ileus had exacerbated (Figure 3). Because the patient could not tolerate oral intake and medications, he was provided nutrition via total parenteral nutrition.

Using a simple suspension method, dabrafenib and trametinib were initiated via the PEG-J tube. Consequently, abdominal pain and nausea improved within a few days of the initial administration. The drainage from the gastric tube decreased markedly, and oral intake was resumed on day 11.

**Figure 4:**
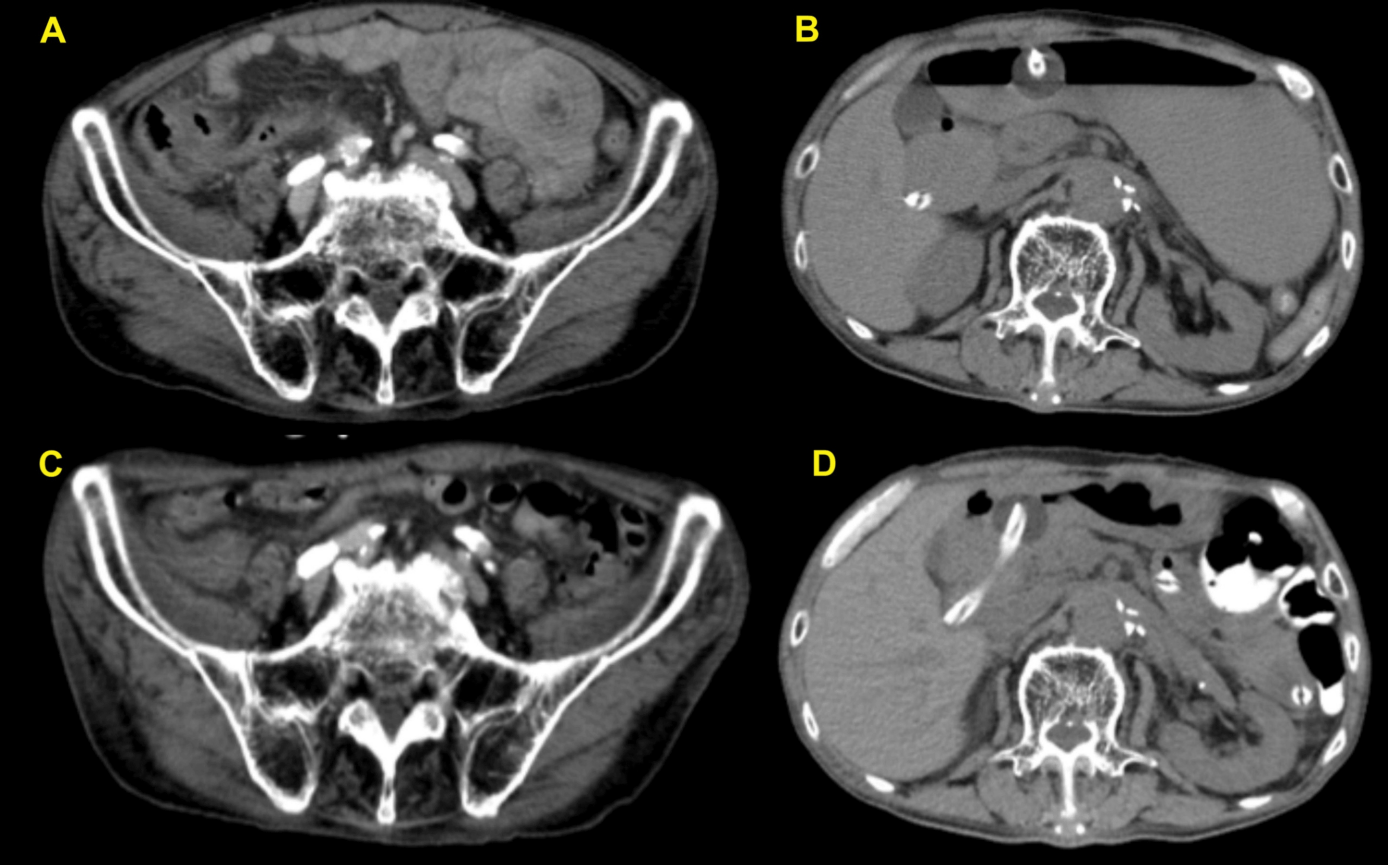
Abdominal CT scan over time (A, B) Before medication, CT showed jejunal intussusception and intestinal ileus. (C, D) On day 12 of medication, CT showed that jejunal intussusception and ileus improved. CT: computed tomography

**Figure 5:**
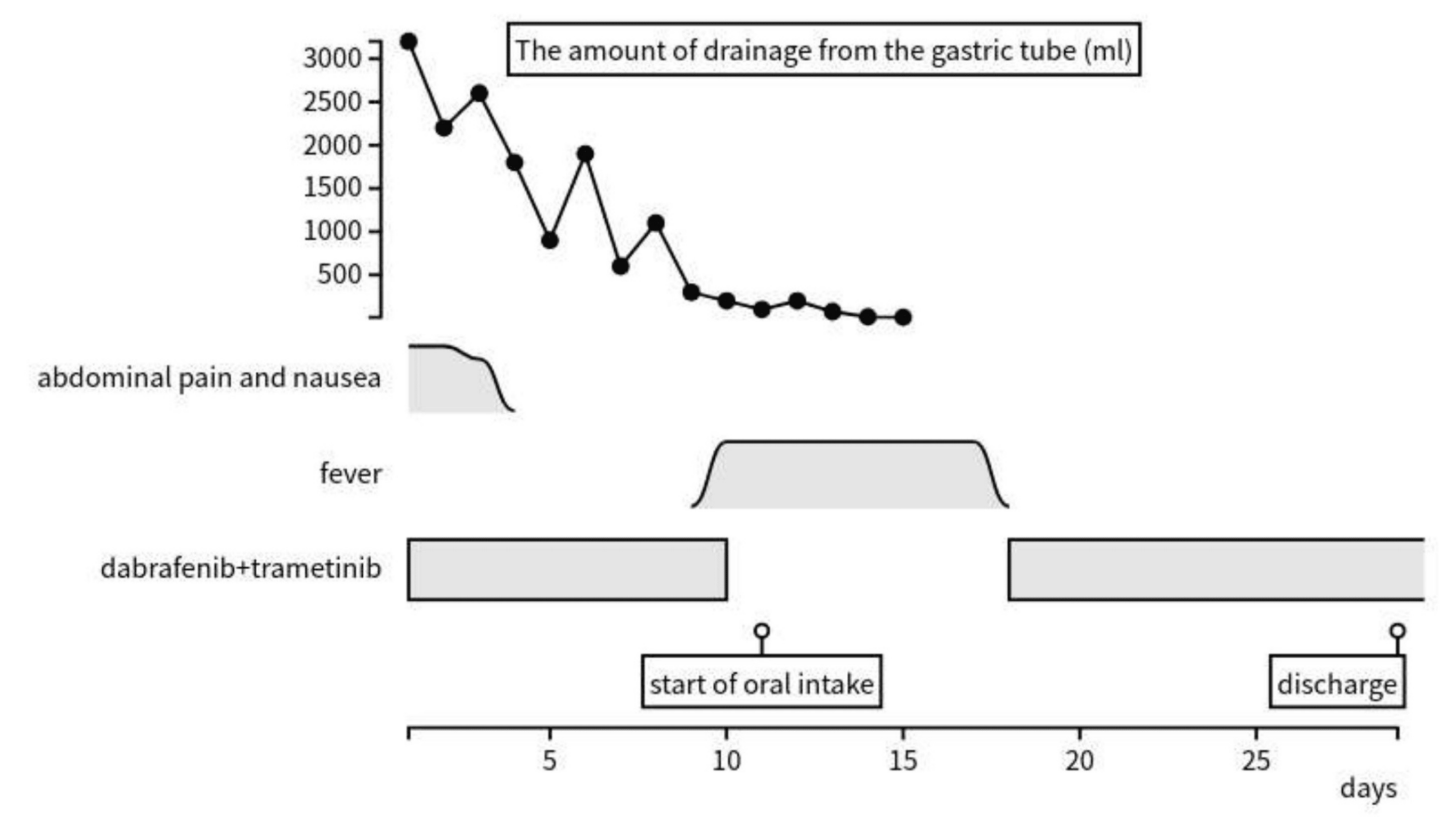
Clinical course from chemotherapy initiation to discharge Abdominal pain and nausea improved within a few days of initial administration. The amount of drainage from the gastric tube decreased markedly.

Between days 10 and 18, the patient developed fever, and chemotherapy was skipped. However, abdominal symptoms did not recur. On day 12, CT imaging showed that intussusception resolved, ileus improved, and the mass size reduced, indicating that chemotherapy was effective (Figure 4). After resuming the medication, chemotherapy was transitioned to oral administration. The patient was discharged from the hospital on day 29. The course from the start of chemotherapy to discharge is shown in Figure 5.

The patient continued chemotherapy while being cared for at home for two months. There was no evidence of tumor regrowth throughout the chemotherapy course. However, the patient's performance status (PS) deteriorated due to the gradual progression of cachexia, which was present before the treatment. Consequently, dabrafenib and trametinib were discontinued on day 102. Palliative care with the goal of treatment switched to the best of supportive care was subsequently initiated. Two months after chemotherapy discontinuation, the patient succumbed to disease progression.

## Discussion

To the best of our knowledge, this is the first case report of the successful treatment of a patient with malignant intestinal obstruction with targeted therapy drugs administered via a PEG-J tube. Targeted therapy drugs could be administered to patients with driver mutations who cannot tolerate oral medication intake due to intestinal obstruction caused by small intestinal metastases, by utilizing feeding tubes, such as PEG-J tubes. Furthermore, therapies targeting driver mutations may be effective for patients with PPC.

Due to the recent advancements in targeted therapy, the survival and quality of life of patients with targetable driver mutations have improved [7]. However, most targeted therapies are administered orally, limiting their use in patients who cannot take medications orally. In previous reports, significant results were observed from administering targeted therapy drugs, including dabrafenib and trametinib, via nasogastric tubes or gastrostomies for patients with dysphagia, anatomical abnormalities, or other digestive disorders. Nevertheless, established evidence is still lacking with regard to the pharmacokinetics, efficacy, and safety of the simple suspension method used in such cases or tablet crushing [7,10-14].

In this case, the patient had an early recurrence of lung cancer postoperatively, with multiple metastases, including to the small intestine, leading to intussusception and ileus. The histopathological findings of the small intestinal tumor were consistent with the preoperative lung tissue, indicating that dabrafenib and trametinib might be effective treatments. However, oral or nasogastric tube drug administration was difficult due to ileus. Moreover, the worsened patient's PS precluded the use of cytotoxic chemotherapy or the expectation of efficacy from immune checkpoint inhibitors.

Therefore, a PEG-J tube was placed distal to the site of the intussusception, enabling the administration of dabrafenib and trametinib while simultaneously decompressing the stomach. Remarkable clinical improvement was observed shortly after treatment initiation. In this case, the drugs were administered using a simple suspension method instead of crushing or decapsulation. We thought that it was appropriate to prevent potential hazards to the medical staff, such as inhalation or skin contact during the drug preparation process; minimize physical changes to the drug, which could impact efficacy; and reduce the risk of tube clogging.

Information on the molecular characteristics of PPC remains limited. Some studies have reported that the most common driver mutations in PPC were Kirsten rat sarcoma viral oncogene homolog (KRAS) mutations (27%), followed by epidermal growth factor receptor (EGFR) (8%), mesenchymal-epithelial transition (MET) (8%), and B-Raf proto-oncogene, serine/threonine kinase (BRAF) (2%) [9]. Previous studies have also highlighted that one of the major factors in treatment failure and drug resistance is intratumoral genomic heterogeneity [15,16]. Many PPCs consist of a combination of sarcomatoid and epithelial components [9]. Considering this complex histology, one case reported an epithelial component positive for an EGFR exon 19 deletion and a mesenchymal component positive for the same EGFR exon 19 deletion along with a T790M mutation, which resulted in poor response to gefitinib [17].

Conversely, Nagano et al. demonstrated that 65% (11/17) of PPC samples shared the same driver mutations across sarcomatoid and epithelial components, indicating a shared clonal origin [9]. In this case, the tissue obtained from the small intestinal tumor biopsy closely resembled the histopathological findings of the preoperative lung tissue, which was consistent with PPC metastasis. Because the patient significantly improved clinically following treatment initiation, BRAF-V600E mutation may have been present in the sarcomatoid and epithelial components of the tumor.

## Conclusions

For patients with NSCLC harboring driver mutations who cannot tolerate medications orally or via a nasogastric tube, administration of targeted therapy drugs through a PEG-J tube represents a viable alternative. However, current data on the efficacy and safety of the administration of targeted drugs via feeding tubes, including PEG-J tubes, are limited and warrant further investigation. Additionally, evidence indicates that the epithelial and sarcomatoid components of PPC may share activating driver mutations, indicating that targeted therapy drugs could be an effective treatment option for patients with PPC with druggable mutations.
